# Porphyrin-polymer nanocompartments: singlet oxygen generation and antimicrobial activity

**DOI:** 10.1007/s00775-017-1514-8

**Published:** 2017-12-07

**Authors:** Angelo Lanzilotto, Myrto Kyropoulou, Edwin C. Constable, Catherine E. Housecroft, Wolfgang P. Meier, Cornelia G. Palivan

**Affiliations:** Department of Chemistry, University of Basel, BPR 1096, Mattenstrasse 24a, 4058 Basel, Switzerland

**Keywords:** Porphyrin, Photosensitizer, Singlet oxygen, Methionine, Polymersomes

## Abstract

**Abstract:**

A new water-soluble photocatalyst for singlet oxygen generation is presented. Its absorption extends to the red part of the spectrum, showing activity up to irradiation at 660 nm. Its efficiency has been compared to that of a commercial analogue (Rose Bengal) for the oxidation of l-methionine. The quantitative and selective oxidation was promising enough to encapsulate the photocatalyst in polymersomes. The singlet oxygen generated in this way can diffuse and remain active for the oxidation of l-methionine outside the polymeric compartment. These results made us consider the use of these polymersomes for antimicrobial applications. *E. coli* colonies were subjected to oxidative stress using the photocatalyst–polymersome conjugates and nearly all the colonies were damaged upon extensive irradiation while under the same red LED light irradiation, liquid cultures in the absence of porphyrin or porphyrin-loaded polymersomes were unharmed.

**Graphical abstract:**

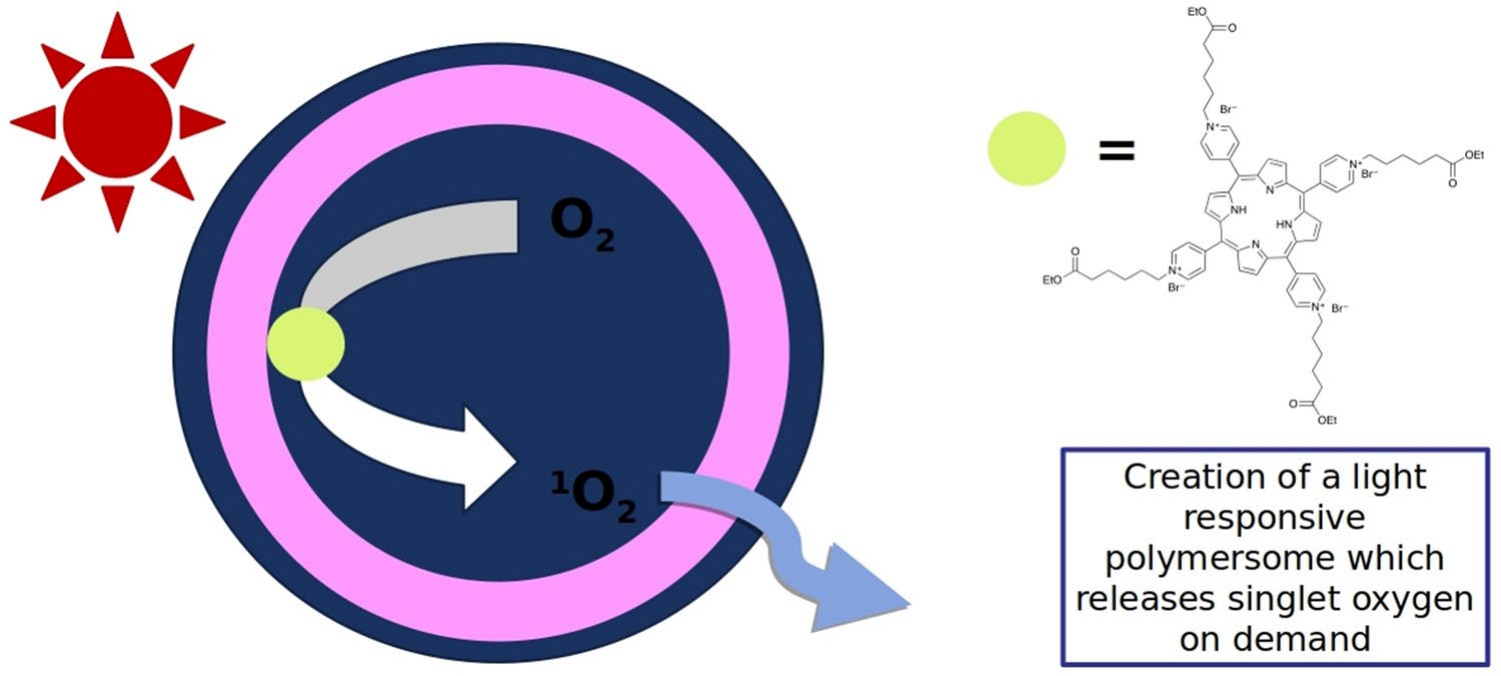

**Electronic supplementary material:**

The online version of this article (10.1007/s00775-017-1514-8) contains supplementary material, which is available to authorized users.

## Introduction

**Scheme 1:**
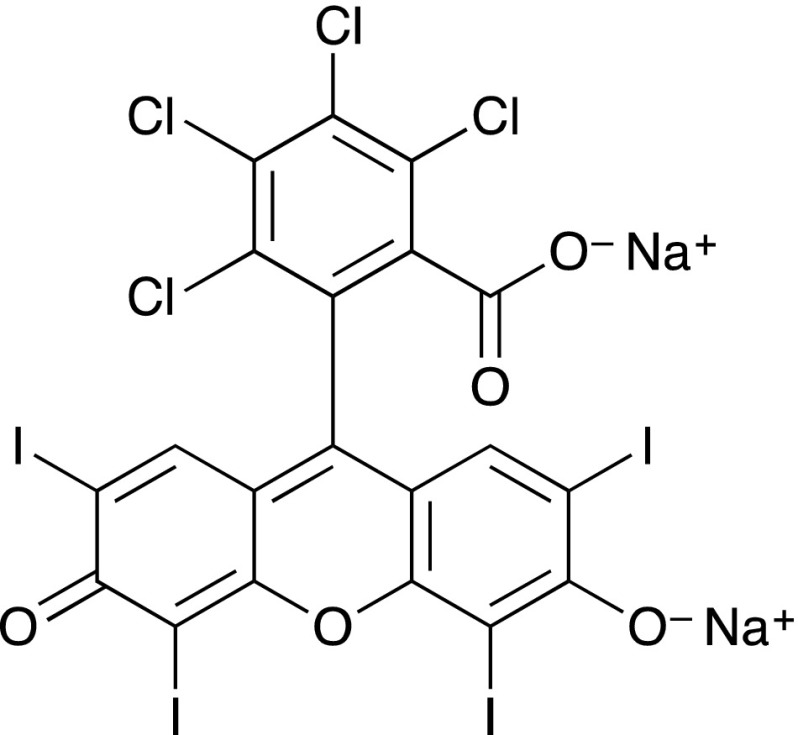
The structure of Rose Bengal

Oxidative stress is associated with a high flux of free radicals and with various pathologic conditions. In contrast, reactive oxygen species (ROS) are also useful in the treatment of tumours and as antimicrobials. The benefit of ROS over conventional approaches with drugs is that the ROS may be generated locally at the target of interest and that the lifetime of the active species is limited, allowing both spatial and temporal control of delivery of the active agent. A variety of photocatalysts generate singlet oxygen upon irradiation in the presence of triplet oxygen. Among them, the most widely used are UV-absorbing compounds including fullerenes [1, 2] naphthalene and anthracene derivatives [3–5] and quinones [6], blue-absorbing flavins [7, 8] and coumarins [9], green-absorbing xanthene derivatives eosin [10, 11], erythrosin [12, 13], fluorescein [13, 14], phloxine [15], Rose Bengal [13] and red-absorbing porphyrins [16–18], phthalocyanines [19] and methylene blue [20, 21]. The D_2_O solution absorption spectrum of Rose Bengal (Scheme 1) has an absorption maximum at 559 nm and acts as a photosensitizer when irradiated with green light. In contrast, porphyrin-based photosensitizers can be triggered by irradiation in the red region of the visible spectrum, despite the dominance in the absorption spectrum of the Soret band at higher energy.

To improve the effect of photosensitizers in the specific cell compartments where they function and to avoid side effects associated with their presence in other regions, a number of carrier systems have been developed. The use of carriers based on nanoassemblies is very appealing because such assemblies (micelles, nanoparticles, polymersomes) can be chemically designed to possess the necessary sizes and properties to be taken up by cells, protect the photosensitizers and release them only under specific conditions related to the cell compartments where singlet oxygen and related ROS production is required [22, 23].

The use of nanoassemblies to host photosensitizers (PS) based both on natural and synthetic polymers provides an efficient and safe approach to photodynamic therapy (PDT) [24, 25] or to generate surfaces with antimicrobial properties [26]. As long as the functionality of the PS remains intact within the nanoassemblies during the photoactivation process, the system can reach its full potential and new opportunities for biomedical applications become accessible. Among the different assemblies, polymer vesicles (or polymersomes) are excellent candidates for this purpose. Their properties can be tuned leading to systems that are mechanically more stable than lipid-based compartments (liposomes). Furthermore, if appropriately selected with respect to the chemical nature of the amphiphilic copolymers, polymersomes are biocompatible and may be stimuli-responsive. The hollow spherical architecture of polymersomes permits the encapsulation of hydrophilic compounds and the insertion of hydrophobic compounds into their membrane [27, 28]. An elegant solution to improve the control of the photosensitizers is not to release them at the desired cell compartments [29, 30], but to ensure that they remain encapsulated in polymersomes allowing one to produce ROS ‘on demand’ upon irradiation [31]. Using this approach, we have shown that encapsulated Rose Bengal conjugated with BSA generates singlet oxygen inside the cavity of polymersomes upon irradiation, and the associated ROS is released in the environment of the polymersomes and is able to induce apoptosis [31, 32]. Reports indicating that porphyrin derivatives have increased intrinsic toxicity upon irradiation in various cell lines [33] encourage the use of nanocarriers to decrease their intrinsic toxicity through encapsulation.

**Scheme 2:**
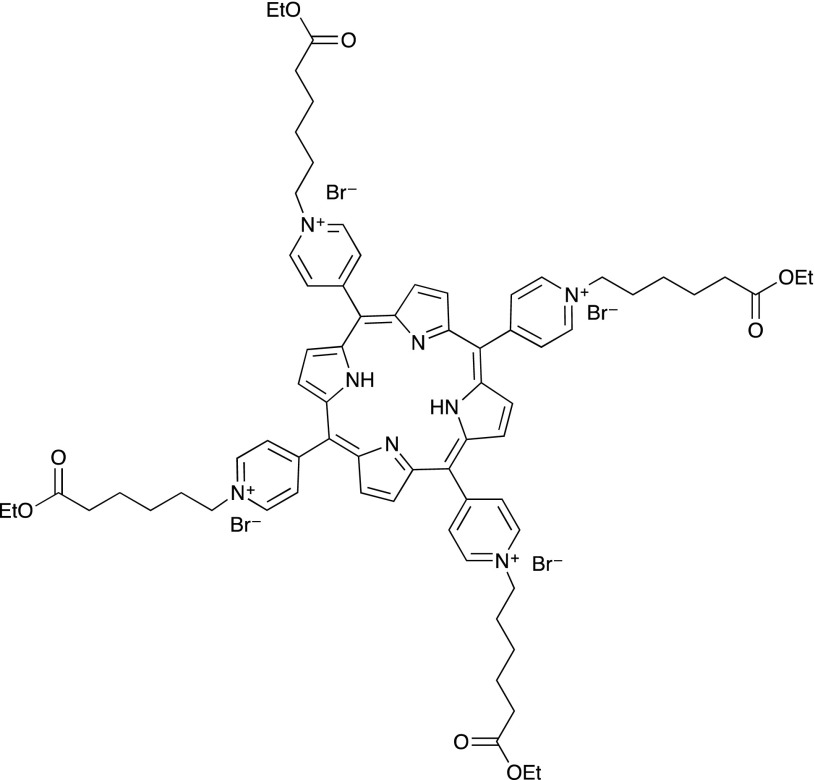
Structure of the photosensitizer TPyCP

In this paper, we demonstrate the preparation of efficient photosensitizer-polymersomes by encapsulation of the porphyrin TPyCP (Scheme 2). A key aspect is the use of a water-soluble porphyrin which is efficient under low energy light conditions even when combined in a robust polymer nanoassembly. Upon irradiation, the porphyrin remains within the aqueous cavity of the polymersome, while the singlet oxygen generated diffuses through the polymer membrane. This results in a nano-system that is safer than direct administration of the porphyrin or a drug delivery system. Water-solubility of the photosensitizer together with its encapsulation within the cavity of the polymersomes are key advantages for further medical applications of our system. We have developed a nano-scale polymersome system, the functions and light responsiveness of which have been evaluated and compared to those of the free porphyrin. Porphyrin-polymersomes have been generated by self-assembly of PMOXA_x_–PDMS_y_–PMOXA_x_, a symmetric amphiphilic C which we have previously used for photosensitizer encapsulation [31, 32]. We also report a biological application of our system by evaluating the TPyCP-loaded polymersomes for their antimicrobial activity against *E. coli*.

## Materials and methods

### General

^1^H and ^13^C NMR spectra were recorded on a Bruker Avance III-400 NMR spectrometer. ^1^H and ^13^C NMR chemical shifts were referenced to residual solvent peaks with respect to *δ*(TMS) = 0 ppm. Solution absorption spectra were recorded using an Agilent Cary 5000 spectrophotometer and ESI mass spectra were recorded using a Shimadzu LCMS-2020 instrument. The LED light source used to activate the photocatalyst was a THORLABS 4-Wavelength High-Power LED Source LED4D067.

### Synthesis of TPyCP

The compound was prepared according to literature [34]. The compounds TPyP, ethyl 6-bromohexanoate and l-methionine were purchased from Sigma-Aldrich and used without further purification.

### Photocatalysis

l-Methionine (200 mg, 1.34 mmol) and TPyCP (0.25 mg, 0.165 μmol, 0.013 mol %) were dissolved in D_2_O (5 mL) in a 5 mL round-bottomed flask. The reaction was repeated using 0.006 mol% catalyst loading: l-methionine (200 mg, 1.34 mmol) and TPyCP (0.12 mg, 0.79 μmol, 0.006 mol%). In each case, the flask was closed with a rubber septum and two needles were inserted through the septum. A pump was connected to one needle to bubble air into the solution; the other was used as an exhaust. The LED lamp was placed so that the light source was perpendicular to the flask ensuring that the whole of the solution was irradiated. Conversion was monitored using ^1^H NMR spectroscopy.

### Kinetics

l-Methionine (63 mg, 0.42 mmol) was dissolved in D_2_O (5 mL) in a 5 mL round-bottomed flask. Either TPyCP (1.3 mg, 0.84 μmol, 0.2 mol%) or Rose Bengal (4.3 mg, 4.2 μmol, 1 mol%) were added to the solution and the flask was capped with a rubber septum and two needles were inserted through the septum. The flask was connected to a pump to force air in through one needle and the second needle was the outlet. The mixture containing Rose Bengal was irradiated at 505 nm and that with TPyCP at 660 nm. Conversion of l-methionine to l-methionine sulfoxide was monitored using ^1^H NMR spectroscopy by comparison of the relative integrals of the resonances of the signals for the Me group adjacent to the sulfur atom (*δ* 2.10 ppm for l-methionine and *δ* 2.70 ppm for l-methionine sulfoxide). Reactions were repeated with different catalyst loadings as detailed in the text. Kinetic studies were also performed with the TPyCP-loaded polymersomes in three different initial concentrations. These results will be discussed in the next section.

### Polymersome preparation

For the formation of the polymersomes, the amphiphilic triblock copolymer PMOXA_34_–PDMS_6_–PMOXA_34_ [35] and prepared as previously described was used. The film rehydration method was followed [36]. Polymer (5 mg) was dissolved in MeOH (1 mL) and dried under vacuum to form a polymer film on the inner bottom surface of a 5 mL glass flask. The polymer film was rehydrated with Tris buffer (50 mM, pH 7.6) at room temperature for 48 h in the dark in the presence or absence of a 50, 100 and 200 μΜ TPyCP solution, respectively. The suspension was then sequentially extruded through 0.2 and 0.1 μm Nucleopore Track-Etch membranes from Whatman using an Avanti Extruder (Avanti Polar Lipids, USA). Any TPyCP left in solution was separated from the polymersomes containing TPyCP by passage through a HiTrap desalting column (Sephadex G-25 Superfine, GE Healthcare, UK) or a 20 cm^3^ in-house prepacked column (Sepharose 2B, Sigma-Aldrich). The polymersomes obtained were characterized by light scattering measurements (LS) and transmission electron microscopy (TEM).

### Transmission electron microscopy

For visualization, 10 μL of a polymersome solution was negatively stained with 2% aqueous uranyl acetate solution, deposited on a carbon-coated copper grid, and then examined with a transmission electron microscope (Philips Morgani 268 D) operating at 80 kV.

### Light scattering

Dynamic (DLS) and static (SLS) light scattering experiments were performed on an ALV (Langen, Germany) goniometer equipped with an ALV He–Ne laser (JDS Uniphase, wavelength *λ* = 632.8 nm). Polymersome emulsions were serially diluted to polymer concentrations ranging from 5 to 0.325 mg/mL, and measured in 10 mm cylindrical quartz cells at angles of 30°–150° and a temperature of 293 ± 0.5 K. The photon intensity autocorrelation function *g* ^2^(*t*) was determined with an ALV-5000E correlator (scattering angles between 30° and 150°). A non-linear decay-time analysis supported by regularized inverse Laplace transform of *g* ^2^(*t*) (CONTIN algorithm) was used to analyse DLS data. The angle-dependent apparent diffusion coefficient was extrapolated to zero momentum transfer (*q* ^2^) using the ALV/Static and dynamic FIT and PLOT 4.31 software. Angle and concentration-dependent SLS data were analysed using Guinier plots. Errors were calculated from the deviation of the fit parameters using the ALV/static and dynamic FIT and PLOT software

### Fluorescence spectroscopy

The fluorescence measurements were carried out on an LS 55 fluorescence spectrometer from Perkin Elmer with a FL Winlab software. Polymersomes loaded with TPyCP were measured in a 1 cm path length quartz cuvette. A wavelength of 424 nm was used to excite in the Soret band and the emission was monitored at 580 nm. Excitation and emission slits were set at 7.5 nm.

### Confocal laser scanning microscopy (CLSM)

CLSM experiments were performed on a confocal laser scanning microscope (Zeiss LSM 880, Carl Zeiss, Jena, Germany) with an Argon/2 laser (*λ* = 488 nm, 30 mW, 10% power output, 0.5% transmission) as the excitation source. A main dichromatic beam splitter (HFT 488/543), and a band pass filter (BP 505–530) were used in all experiments. The images were recorded with a water immersion objective (C-Apochromat 40×/1.2 W).

### Bacterial assays

Aliquots of 5 μL taken from a stock *E. coli* colony were dispersed into 15 mL of lysogeny broth (LB) in a 50 mL falcon tube. The suspension was shaken at 180 rpm in an incubator overnight at 37 °C, with the cup not entirely closed to allow molecular oxygen to diffuse and reach the bacteria. The culture was then concentrated by centrifugation at 10,000*g* for 10–15 min. The supernatant was removed and the culture was resuspended in 15 mL of phosphate buffered saline solution (PBS), centrifuged again to remove the remaining media and resuspended for the last time in 15 mL of PBS containing 1% tryptic soy broth (TSB). The bacterial culture was serially diluted (10^−1^–10^−5^ CFU/10 μL, CFU being colony forming units) in a 24-well plate. In the last liquid culture, which corresponds to the desired dilution, 200 μL of an aqueous TPyCP solution or a solution with polymersomes containing TPyCP were added. The 24-well plate was kept in the dark until the exposure to LED irradiation started. Liquid cultures were taken and placed into LB-Agar plates after 0, 30, 120, 240 and 360 min of irradiation. The plated cultures were incubated overnight at 37 °C and the colonies formed counted. As a control, the same series of experiment were performed in the absence of TPyCP. Furthermore, a so-called dark control was performed simultaneously: an *E. coli* culture in the presence of TPyCP was kept in the dark by means of aluminum foil wrapping. Three independent experiments were run, and for each three replicates were plated. Both controls were carried out during each experiment. All the *E. coli* essays were carried out in a sterile environment.

### Live/Dead^®^ staining for microscopy

For this assay, the Live/Dead^®^ BacLight (https://assets.thermofisher.com/TFS-Assets/LSG/manuals/mp07007.pdf). Bacterial Viability Kit for microscopy has been used. Briefly, the *E. coli* bacteria liquid cultures were grown as described previously, with the difference that instead of PBS, aqueous 0.85% NaCl (0.85 g per 100 mL) was used as the suspension buffer. After illumination with a red LED light at 660 nm, the cultures were stained with a 1:1 mixture of SYTO 9 (https://assets.thermofisher.com/TFS-Assets/LSG/manuals/mp07572.pdf), which belongs to the family of SYTO dyes and is a cell-permeant nucleic acid stain, and propidium iodide dyes using 3 μL of 72 nM stain per 1 ml of sample, and then incubated for 10 min at room temperature. Bacteria with intact cell membranes (considered alive) stained fluorescent green, whereas bacteria with damaged membranes (considered dead) stained fluorescent red. The excitation/emission maxima for these dyes are in the range 480–500 nm for SYTO 9 and 490–635 nm for propidium iodide. The background remains virtually non-fluorescent. The results were analysed by CLSM.

## Results and discussion

### Photosensitizer

**Fig. 1:**
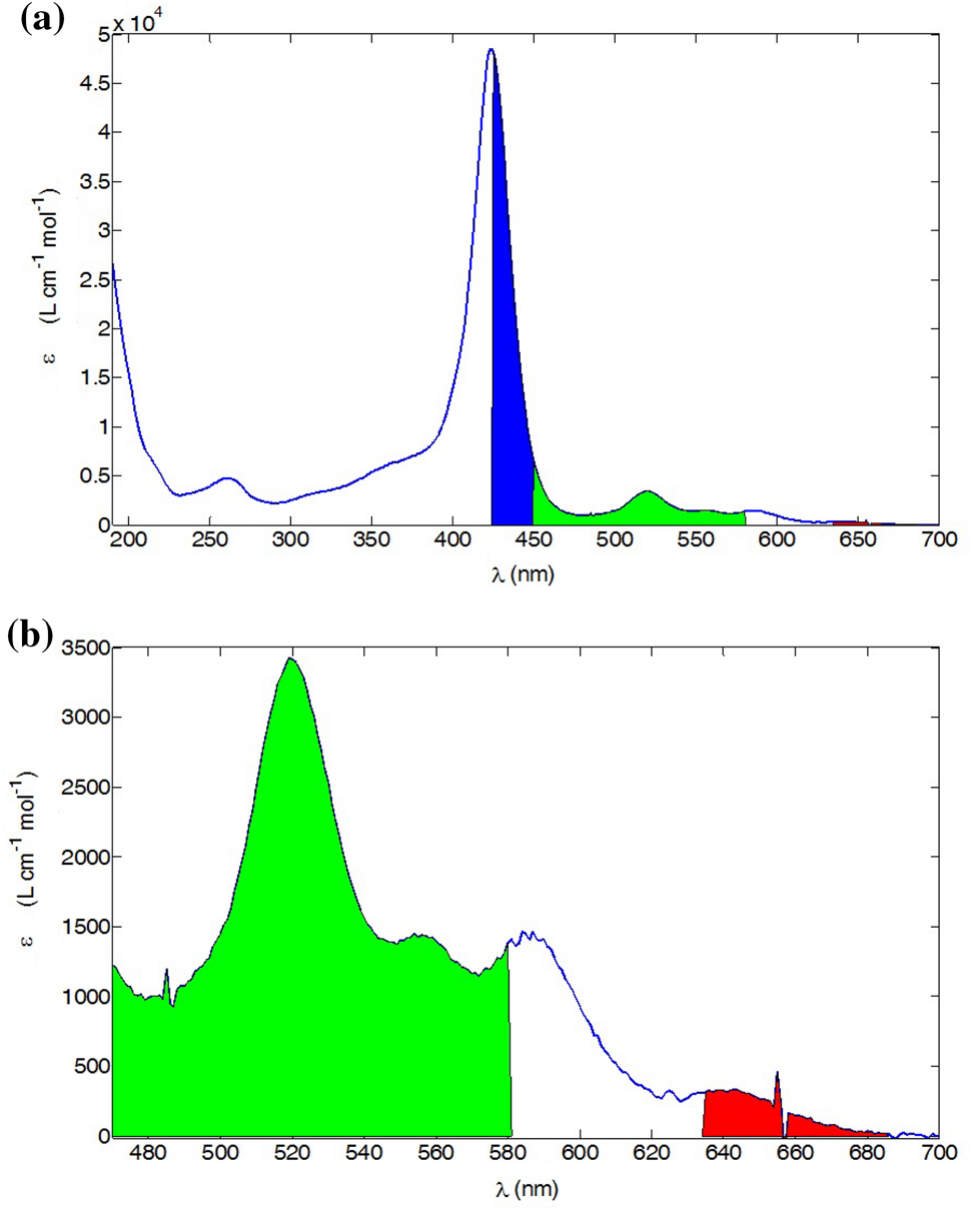
**a** The absorption spectrum of TPyCP (4 μM in D_2_O). The coloured areas show the overlap of the absorption with the emission bands of the LED used for irradiation. **b** Expansion of the low energy part of the spectrum

Water-soluble porphyrins may be obtained through functionalization of the phenyl groups of tetraphenylporphyrin (TPP) with carboxylic acid or sulfonate groups or by *N*-alkylation of the otherwise water-insoluble 5,10,15,20-tetra(pyridin-4-yl)porphyrin (TPyP). We chose the latter approach, since *N*-alkylation of TPyP allows the introduction of a variety of functionalities at the termini of the alkyl chains. In the present investigation, an ester functionality was chosen, giving the potential for further derivatization at a later stage. The compound TPyCP (Scheme 2) was prepared by treatment of TPyP with an excess ethyl 6-bromohexanoate in boiling DMF according to the previously reported method [34]; the crude product was precipitated by addition of Et_2_O and then recrystallized twice from ethanol. The NMR spectrum of the product was consistent with the literature data [34]. Figure 1 shows the solution absorption spectrum of TPyCP in D_2_O which shows all the hallmarks of a free-base porphyrin: an intense Soret band with *λ*_max_ = 424 nm (*ε*_max_ = 48,500 dm^3^/mol/cm) and four *Q* bands at lower energy (*λ*_max_ = 519, 555, 586 and 640 nm, *ε*_max_ = 3400, 1400, 1400 and 300 dm^3^/mol/cm, respectively). The absorptions at higher energy (< 400 nm) arise from the alkylpyridinium units.

**Fig. 2:**
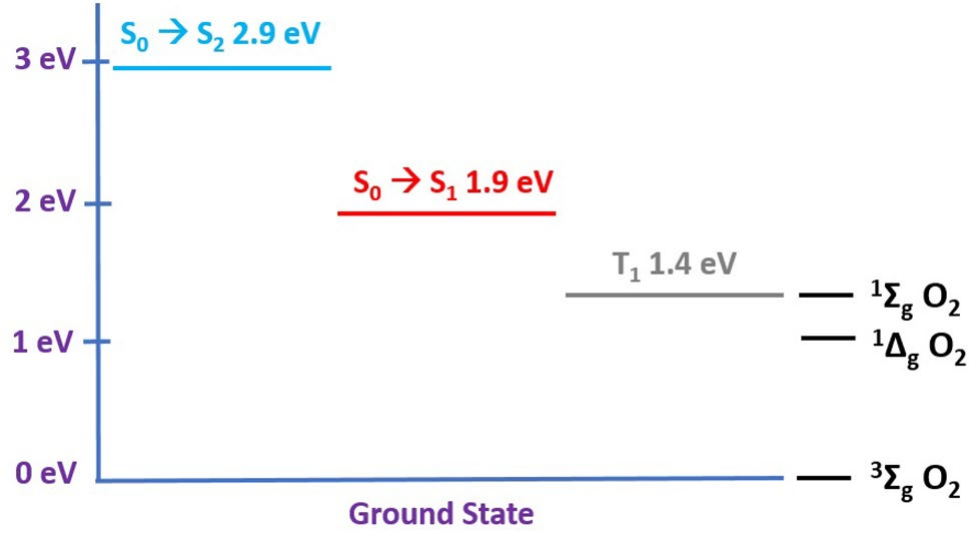
Excited state energy levels for TPyCP compared to the ground and low-lying excited states of O_2_. The S_1_ electronic state can be populated by means of both green and red LED light by virtue of its extended absorption (vibrational structure)

The LED used to activate the photocatalyst TPyCP operated at three wavelengths: 470, 505 and 660 nm and the overlap of these excitation bands with the absorption spectrum of TPyCP is highlighted in Fig. 1. Figure 2 summarizes the relevant energy levels of TPyCP compared to the ground and low-lying excited states of O_2_ [37]. The energy of the TPyCP T_1_ state is taken from the literature data for the compound 5,10,15,20-tetra(*N*-methylpyridin-4-yl)-21*H*,23*H*-porphyrin tetrachloride [38]; the introduction of a long alkyl chain in place of a methyl group is not expected to affect the photochemistry. The triplet state energy is expected to be conserved in the two compounds and the literature value can be used. Irradiation at 470 nm populates the S_2_ state of TPyCP, whilst irradiation at 505 or 660 nm populates the S_1_ state. After population of the lower energy S_1_ state by use of the red LED, an intersystem crossing leads to TPyCP in the T_1_ state. In fluid solution, T_1_ is non-emissive and lives long enough (lifetime = 170 μs) to be quenched by molecular oxygen [38]. Energy transfer from the T_1_ state of TPyCP to molecular O_2_ can occur to either of the ^1^Σ_g_ or ^1^Δ_g_ excited states since both are of appropriate energy (Fig. 2). ^1^Σ_g_ quickly deactivates to ^1^Δ_g_ and singlet oxygen reactivity derives from this state [39]. Thus, reference to singlet oxygen in the subsequent discussion refers to ^1^Δ_g_^1^O_2_.

### Methionine substrate and a comparison with Rose Bengal sensitization

**Scheme 3:**
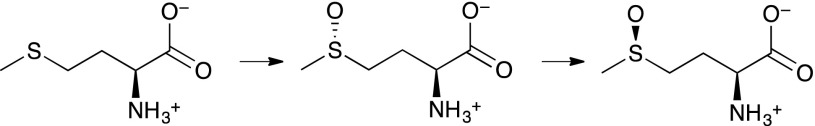
Conversion of l-methionine to the diastereoisomeric pair of l-methionine sulfoxides. Atom labels are for NMR spectroscopic assignments

The substrate chosen for the photocatalytic oxidation investigation was the amino acid, l-methionine [40]. Its selective oxidation to the sulfoxide represents a model for the oxidation of methionine residues in proteins (see for example: 41–44]). An aqueous solution of l-methionine containing 0.06 mol% of TPyCP was irradiated with red light (*λ*_exc_ = 660 nm) and the reaction mixture was monitored by ^1^H NMR spectroscopy. Complete and selective conversion of l-methionine to sulfoxide (Scheme 3) was achieved in 61 h. The ^1^H and ^13^C NMR spectra of the product (Figs. S1 and S2) were consistent with those of pristine l-methionine sulfoxide [45]. A comparison of the spectra of l-methionine and l-methionine sulfoxide confirmed the generation of a second stereogenic centre (Figs. S1 and S2) with ^13^C NMR resonances for atoms C^2^ and C^5^ (see Scheme 3) each showing the presence of the expected two diastereoisomers. The ESI–MS of the product showed a peak at *m/z* 165.94 corresponding to [M+H]^+^, M being l-methionine sulfoxide.

**Fig. 3:**
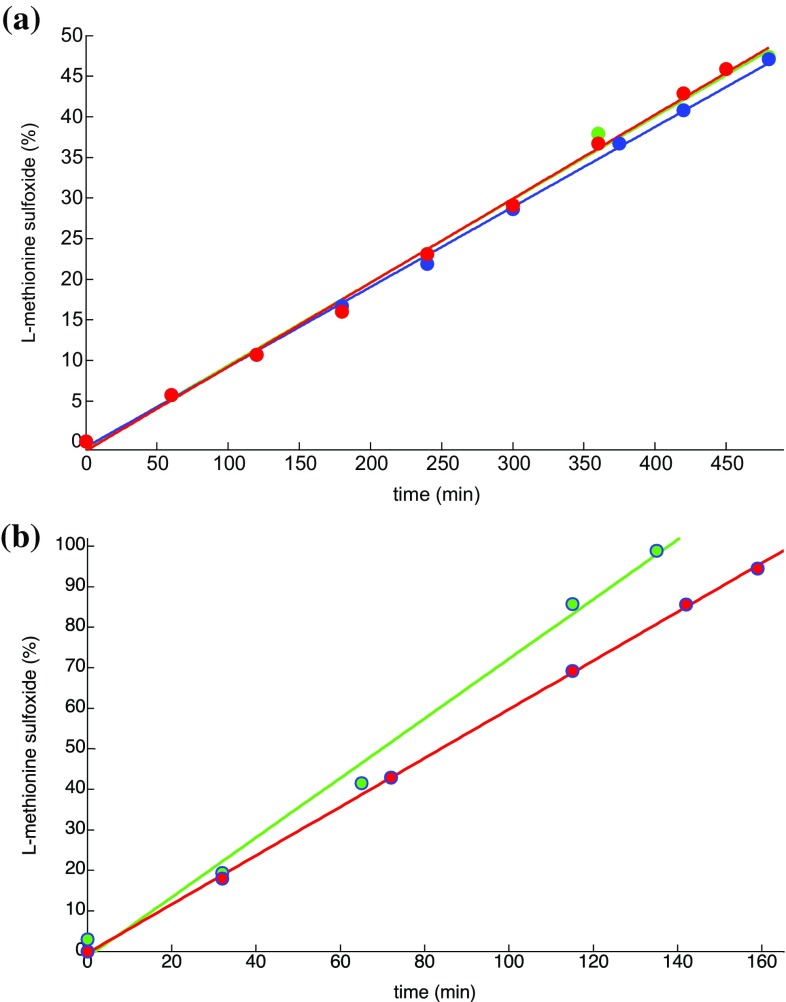
**a** l-Methionine conversion (given as % on the ordinate) to the diastereoisomeric l-methionine sulfoxides in the presence of 0.06 mol% TPyCP. 470 nm irradiation (blue), 505 nm irradiation (green), 660 nm irradiation (red). **b** Conversion of a 0.42 mmol l-methionine solution in D_2_O in presence of 0.2 mol% TPyCP (red) or 1 mol% Rose Bengal (green). Irradiation was performed at 505 nm for Rose Bengal or at 660 nm for TPyCP. Data points (circles) and linear best fit (straight lines), *r* ^2^ = 0.9915 for Rose Bengal and 0.9872 for TPyCP

This preliminary study confirmed that even though light absorption by TPyCP at 660 nm is low (< 1000 L/cm/mol, Fig. 1), a 0.06 mol% catalytic loading is sufficient to realize complete selective oxidation within 61 h. To confirm that the rate of reaction was not enhanced by irradiating the sample with higher energy light, the reaction was repeated using *λ*_exc_ = 470, 505 and 660 nm. For each reaction, 0.35 mmol of l-methionine in 5 mL D_2_O containing 0.06 mol% of TPyCP was used, and the reaction was followed by ^1^H NMR spectroscopy. Despite the differences in overlap of the absorption spectrum of TPyCP with the LED excitation bands shown in Fig. 1, there was no difference in the rate of the reaction (Fig. 3). This can be rationalized in terms of the energy level diagram shown in Fig. 2. Recall that irradiation at 470 nm populates the S_2_ state of TPyCP, while with *λ*_exc_ = 505 or 660 nm, the S_1_ state is directly populated. The first process after excitation, regardless of the excitation wavelength, is the fast (according to Kasha’s rule) relaxation to lower lying excited states and ultimately to T_1_. From T_1_, oxygen sensitization occurs independent of the wavelength of the excitation light, and so the photocatalysis yields at any given time will be the same (Fig. 3).

We next carried out a comparative investigation of the kinetics of the selective oxidation of l-methionine using TPyCP or the commercially available Rose Bengal as photosensitizer. The aim of this study was to determine whether use of the TPyCP could be advantageous over the commercial and widely used Rose Bengal. The data in Fig. 3a confirmed that TPyCP acts with equal efficiency upon irradiation at 505 or 660 nm. In contrast, Rose Bengal possesses no absorption at 660 nm and, as expected, no conversion of l-methionine to its sulfoxides was observed upon irradiating at this wavelength. For the kinetic experiments, a D_2_O solution of l-methionine (0.42 mmol) was irradiated in the presence of TPyCP (0.2 mol%) or Rose Bengal (1 mol%) with *λ*_exc_ = 660 or 505 nm, respectively. The reaction was monitored by ^1^H NMR spectroscopy and the results are shown in Fig. 3b. Both photocatalysts lead to complete conversion of l-methionine to a 1:1 ratio of both diastereoisomers of l-methionine sulfoxide, and although Fig. 3b might suggest that the reaction with Rose Bengal is faster; it is significant that TPyCP is present at 0.2 mol% compared to 1 mol% of Rose Bengal.

**Fig. 4:**
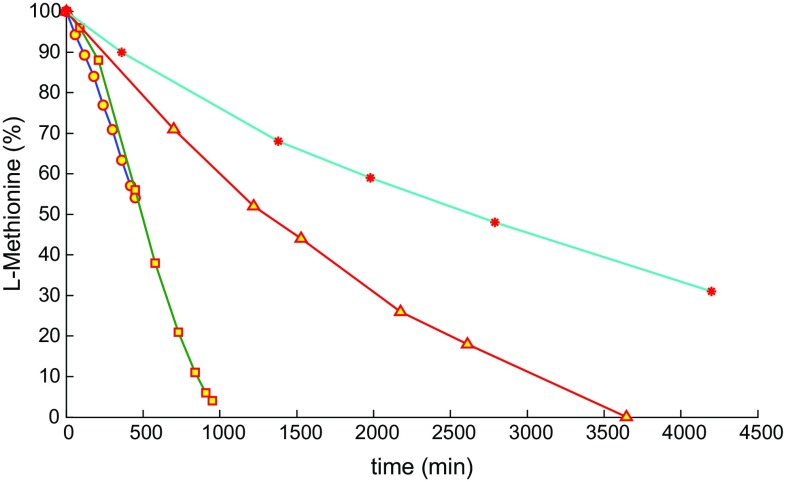
l-Methionine (270 mM in D_2_O) conversion for a TPyCP concentration of 150 μM (0.055 mol%) (circles), 67 μM (0.024 mol%) (squares), 34 μM (0.013 mol%) (triangles) and 17 μM (0.006 mol%) (filled red circles) (*λ*_exc_ = 660 nm)

**Scheme 4:**
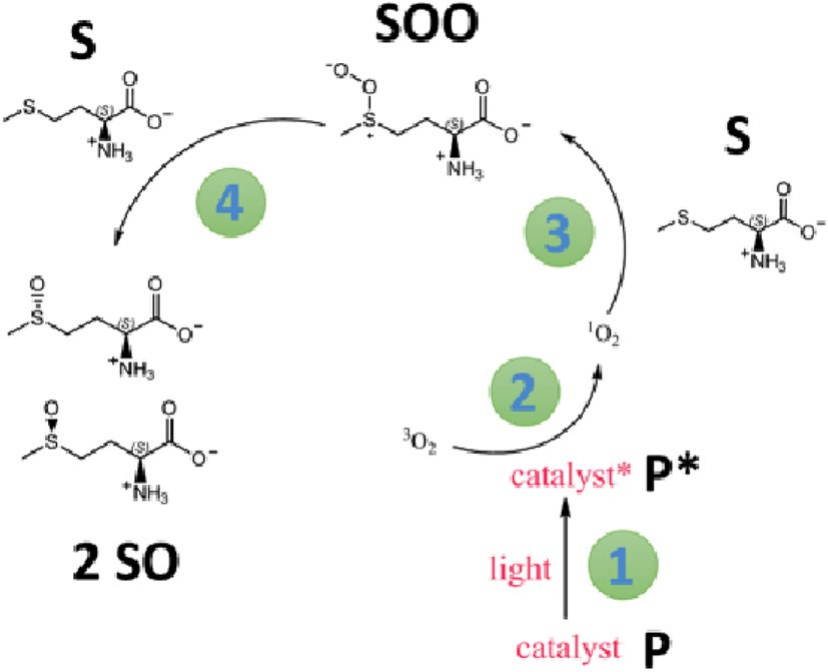
Proposed mechanism for the photocatalytic oxidation of methionine (based on scheme in Ref. [40])

We next varied the catalyst loading and depending on the photocatalyst concentration, either an exponential or linear regime was observed (Fig. 4). Keeping the methionine concentration at 270 mM, for a porphyrin concentration of 150 and 67 μM, a near-linear correlation of conversion to reaction time is observed. If the photocatalyst concentration is either of 34 or 17 μM, an exponential decay is observed. The addition of oxygen to l-methionine and subsequent formation of diastereoisomeric l-methionine sulfoxides has been studied in the past [40, 46] and the mechanism shown in Scheme 4 has been proposed.

The scheme can be summarized, and made more general, by writing Eqs. 1–4 in which P and P* are the porphyrin catalyst in its ground and excited state, respectively, ^3^O_2_ and ^1^O_2_ are molecular oxygen in its triplet ground and singlet excited state, respectively; S, SOO and SO are defined in Scheme 4.

1 $${\text{P}} + hv\mathop \to \limits^{{ k_{1 } }} {\text{P}}^{*}$$

2 $${\text{P}}^{*} +^{3}{\text{O}}_{2} \mathop \to \limits^{{ k_{2 } }} {} ^{1} {\text{O}}_{2}$$

3 $${\text{S}} + ^{1} {\text{O}}_{2} \mathop \to \limits^{{ k_{3 } }} {\text{SOO}}$$

4 $${\text{SOO}} + {\text{S}}\mathop \to \limits^{{ k_{4 } }} 2 {\text{SO}}$$

From Eqs. 1–4, we can derive Eqs. 5–9 for species in the catalytic cycle.

5 $$\frac{{{\text{d}}[{\text{P}}^{ *} ]}}{{{\text{d}}t}} = k_{1} - k_{2} [^{3} {\text{O}}_{2} ][{\text{P}}^{*} ]$$

6 $$\frac{{{\text{d}}[^{1} {\text{O}}_{2} ]}}{{{\text{d}}t}} = k_{2} [^{3} {\text{O}}_{2} ][{\text{P}}^{*} ] - k_{3} [{\text{S}}][^{1} {\text{O}}_{2} ]$$

7 $$\frac{{{\text{d}}[{\text{S}}]}}{{{\text{d}}t}} = - k_{3} [{\text{S}}][^{1} {\text{O}}_{2} ] - k_{4} [{\text{SOO}}][{\text{S}}]$$

8 $$\frac{{{\text{d}}[{\text{SOO}}]}}{{{\text{d}}t}} = k_{3} [^{1} {\text{O}}_{2} ][{\text{S}}] - k_{4} [{\text{SOO}}][{\text{S}}]$$

9 $$\frac{{{\text{d}}[{\text{SO}}]}}{{{\text{d}}t}} = 2k_{4} [{\text{SOO}}][{\text{S}}]$$

Experimentally, it is not possible to detect SOO. It does not accumulate over the course of the reaction and we may therefore, apply the steady-state approximation for this intermediate (Eqs. 10, 11).

10 $$\frac{{{\text{d}}[{\text{SOO}}]}}{{{\text{d}}t}} = k_{3} [^{1} {\text{O}}_{2} ]\left[ {\text{S}} \right] - k_{4} \left[ {\text{SOO}} \right]\left[ {\text{S}} \right] = 0$$

11 $$[{\text{SOO}}] = \frac{{k_{3} [^{1} {\text{O}}_{2} ]}}{{k_{4} }}$$

Equation 11 requires that SOO does not accumulate if k_4_ ≫ k_3_. Substituting Eq. 11 into Eq. 7 leads to Eq. 12, and hence, Eq. 13.

12 $$\frac{{{\text{d}}[{\text{S}}]}}{{{\text{d}}t}} = - 2k_{3} [{\text{S}}][^{1} {\text{O}}_{2} ]$$

13 $$[{\text{S}}] = {\text{e}}^{{ - 2k_{3} [^{1} {\text{O}}_{2} ]t}}$$

From Eq. 13, it follows that the consumption of l-methionine depends exponentially the supply of oxygen (represented by *k*_3_) and the concentration of singlet oxygen. The latter dependency can be eliminated if we consider the experimental setup. Triplet oxygen, ^3^O_2_, is introduced into the reaction vessel by means of a pump, which keeps the concentration of ^3^O_2_ constant at the saturation level of the solvent. In addition, the concentration of the catalyst in the excited state (P*) is also constant. This is due to the fact that the concentration used is such that the absorbance of the reaction mixture at the LED *λ*_exc_ = 660 nm is 0.09 ([catalyst] = 150 μmol dm^−3^, path length = 2.5 cm, *ε* = 200 dm^3^/mol/cm) which converts to 19% of the excitation light being absorbed as it travels through the reaction flask. It is true that, once excited, P is in the lowest singlet excited state, whereas it is the lowest triplet excited state that is able to transfer energy to molecular oxygen. Due to intersystem crossing (ISC) which is a unimolecular process, P* relaxes to this state, which constitutes the active state of the catalyst. We have no reason to assume any of those processes (light absorption in non-saturated conditions and ISC) are time dependent. Furthermore, the extent of the energy transfer to ground state molecular oxygen is a property of a given couple of molecules, and therefore, time independent as well. The latter two considerations allow us to simplify Eq. 12 by applying the steady-state approximation to ^1^O_2_ (Eqs. 14, 15). This species is the product of the reaction between P* and ^3^O_2_, concentrations of which are constant over time.

14 $$\frac{{{\text{d}}[^{1} {\text{O}}_{2} ]}}{{{\text{d}}t}} = k_{2} [^{3} {\text{O}}_{2} ][{\text{P}}^{ *} ] - k_{3} [{\text{S}}][^{1} {\text{O}}_{2} ] = 0$$

15 $$[^{1} {\text{O}}_{2} ] = \frac{{k_{2} [^{3} {\text{O}}_{2} ][{\text{P}}^{ *} ]}}{{k_{3} [{\text{S}}]}}$$

Substituting Eq. (15) into Eq. (12) gives Eq. 17 in which [S]_0_ is the initial concentration on l-methionine. Equation 17 shows a linear relationship between [S] and *t*. All of the other dependencies are eliminated.

16 $$\frac{{{\text{d }}[{\text{S}}]}}{{{\text{d}}t}} = - 2k_{2} [^{3} {\text{O}}_{2} ][{\text{P}}^{ *} ]$$

17 $$[{\text{S}}] = [{\text{S}}]_{0} - 2k_{2} [^{3} {\text{O}}_{2} ][{\text{P}}^{ *} ]t$$

### Photosensitization within polymersomes: PMOXA–PDMS–PMOXA assemblies with and without TPyCP

**Fig. 5:**
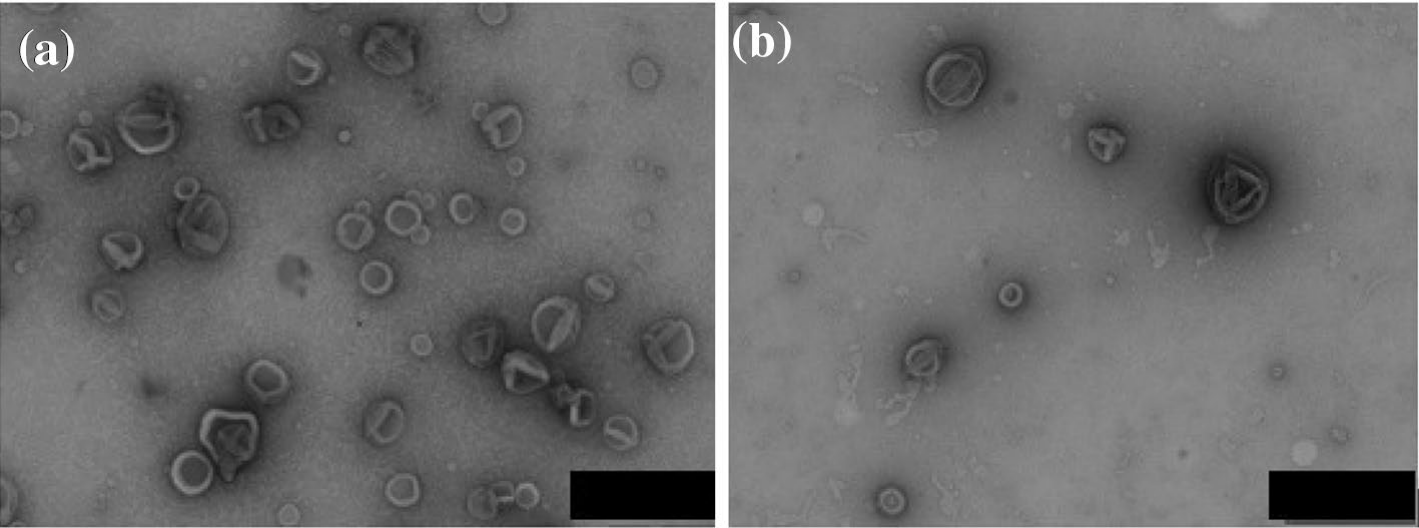
TEM micrographs of polymersomes **a** without TPyCP and **b** with encapsulated TPyCP. Scale bars are 200 nm

The 3D-assemblies resulting from the self-assembly of PMOXA_6_–PDMS_34_–PMOXA_6_ copolymers with and without porphyrin were characterized by a combination of TEM and light scattering. TEM micrographs indicate that spherical assemblies with radii of around 100 nm form in both the presence and absence of TPyCP, and also that a second population of spherical assemblies with a significantly smaller size is formed (Fig. 5a, b). To establish the morphology of the 3D-assemblies, we used static light scattering (SLS) to determine the radius of gyration (*R*_g_), and dynamic light scattering (DLS) to give the hydrodynamic radius (*R*_h_) of the self-assembled objects and calculate the ratio *R*_g_/*R*_h_ (*ρ*-parameter). Copolymers in both the presence and absence of TPyCP self-assembled in spherical supramolecular assemblies with *R*_g_ ~ 100 nm, and ρ values ranging from 0.91 up to 1.08. This range is close to 1, the value characteristic of a hollow morphology (Table 1) [47]. The slight deviation of *ρ* from 1 can be explained by the presence of the second population of small spherical nano-objects, with sizes characteristic of micelles, in agreement with the TEM micrographs. Since the micelles cannot host TPyCP due to the hydrophilic character of the photosensitizer, we can neglect this population in our discussion of photosensitizer-loaded compartments. In addition, an increase of the amount of TPyCP intended to improve the encapsulation efficiency, affected neither the size nor the morphology of the polymersomes, supporting the optimization of the system (Fig. S3).

**Table 1:** Light scattering data for supramolecular assemblies without and with TPyCP

	DLS/SLS		*R*_g_/*R*_h_
	*R*_h_ (nm)	*R*_g_ (nm)	
Polymersomes empty	97	95	0.97
Polymersomes with TPyCP 50 μΜ	101	99	0.98
Polymersomes with TPyCP 100 μΜ	95	103	1.08
Polymersomes with TPyCP 200 μΜ	108	92	0.91

The stated concentration is the initial value

### Encapsulation of TPyCP into polymersomes

**Fig. 6:**
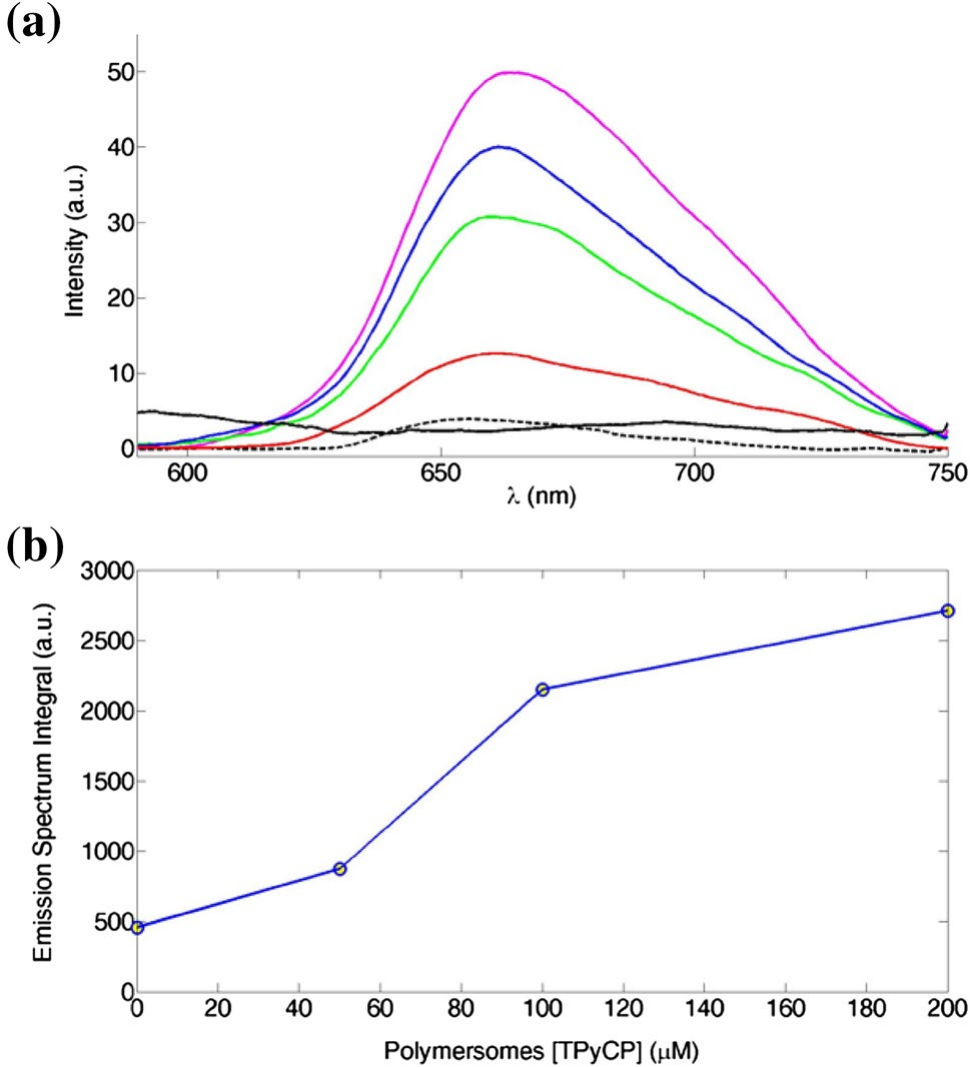
**a** Emission spectra of the free TPyCP and TPyCP-loaded polymersomes: free TPyCP 200 μΜ in Tris buffer (magenta line), TPyCP in polymersomes with an initial concentration of 200 μΜ (blue line), TPyCP in polymersomes with an initial concentration of 100 μΜ (green line), TPyCP 50 μΜ in polymersomes with an initial concentration of 50 μΜ (red line), empty polymersomes (black line), second fraction from the purification of 200 μΜ TPyCP in polymersomes (dashed black line). **b** Integral of the emission spectra for the porphyrin-loaded polymersomes as a function of the initial concentration of TPyCP

Fluorescence spectroscopy was used to determine whether the water-soluble TPyCP present in the rehydration buffer of the copolymer film was encapsulated inside the polymersomes during the self-assembly process (Fig. 6). We focused on the emission peak at 660 nm instead of the absorption peak at 424 nm, because fluorescence is a more sensitive technique than absorption and TPyCP is present at micromolar concentration. The fluorescence intensity of a 200 μΜ TPyCP solution in aqueous Tris buffer was higher than that of the porphyrin-loaded polymersomes after purification, as expected since a fraction of the photosensitizer is not encapsulated during the self-assembly process. We successfully removed the non-encapsulated TPyCP on a Sephadex column and verified that the fraction which eluted after the polymersomes had negligible fluorescence. This is consistent with the visual observation that during the purification process, the initial sample solutions were dark yellow (the characteristic colour of TPyCP at this concentration), while the collected fractions were colourless. As expected, the lower the initial porphyrin concentration, the lower the fluorescence signal associated with the encapsulated photosensitizer: polymersomes with an initial TPyCP concentration of 50 μΜ were barely fluorescent. Empty polymersomes were not fluorescent.

### Photo-activation of photosensitizer within polymersomes

**Fig. 7:**
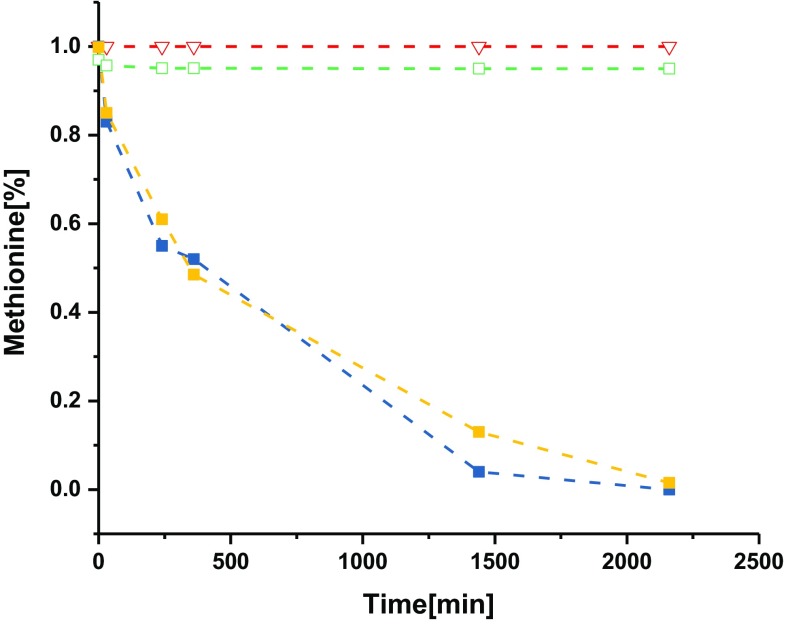
l-Methionine conversion to (*R*)/(*S*)-sulfoxide by free TPyCP (67 μΜ in D_2_O, blue squares), TPyCP-loaded polymersomes with an initial porphyrin concentration of 200 μM (yellow squares), free methionine in solution (red empty triangles) and empty polymersomes incubated with 200 μM TPyCP and then purified (green empty squares) (*λ*_exc_ = 660 nm)

We next focused attention on the selective oxidation of l-methionine using TPyCP-loaded polymersomes to investigate whether the encapsulation of the sensitizer affected the kinetics of the reaction (Fig. 7). Once singlet oxygen is photo-generated inside the inner cavity of a polymersome, it can diffuse through the polymer membrane, as established for PMOXA–PDMS–PMOXA amphiphilic copolymer based polymersomes [32]. Therefore, we expect only the l-methionine in the outer environment (at a concentration of 270 mM in D_2_O) to be oxidized. To confirm that the purification column removed excess TPyCP, we tested a suspension which contained empty polymersomes mixed with 200 μΜ TPyCP for 48 h and then purified under the same conditions as those of the porphyrin-containing polymersomes. In addition, a 150 μΜ l-methionine solution in D_2_O was exposed to molecular oxygen to confirm that self-oxidation of l-methionine did not occur. A system comprising TPyCP-loaded polymersomes with the highest initial concentration of the porphyrin (blue squares in Fig. 7) was the most efficient in terms of singlet oxygen generation and conversion from l-methionine to both diastereoisomeric forms of l-methionine sulfoxide. Both the free porphyrin and the porphyrin-loaded polymersomes successfully converted l-methionine to l-methionine sulfoxides in an exponential manner that reached 100% conversion after 26 h of irradiation. As expected, no oxidation of l-methionine was observed in the presence of empty polymersomes. Similarly, when porphyrins were added to a D_2_O solution containing polymersomes and then the system was purified, i.e., TPyCP present on the outside was removed, no conversion of l-methionine was observed upon irradiation. The results are consistent with singlet oxygen induced a non-stereoselective conversion of l-methionine to l-methionine sulfoxides occurring only with porphyrins encapsulated inside polymersomes.

### Biological evaluation of TPyCP in solution and within polymersomes

**Fig. 8:**
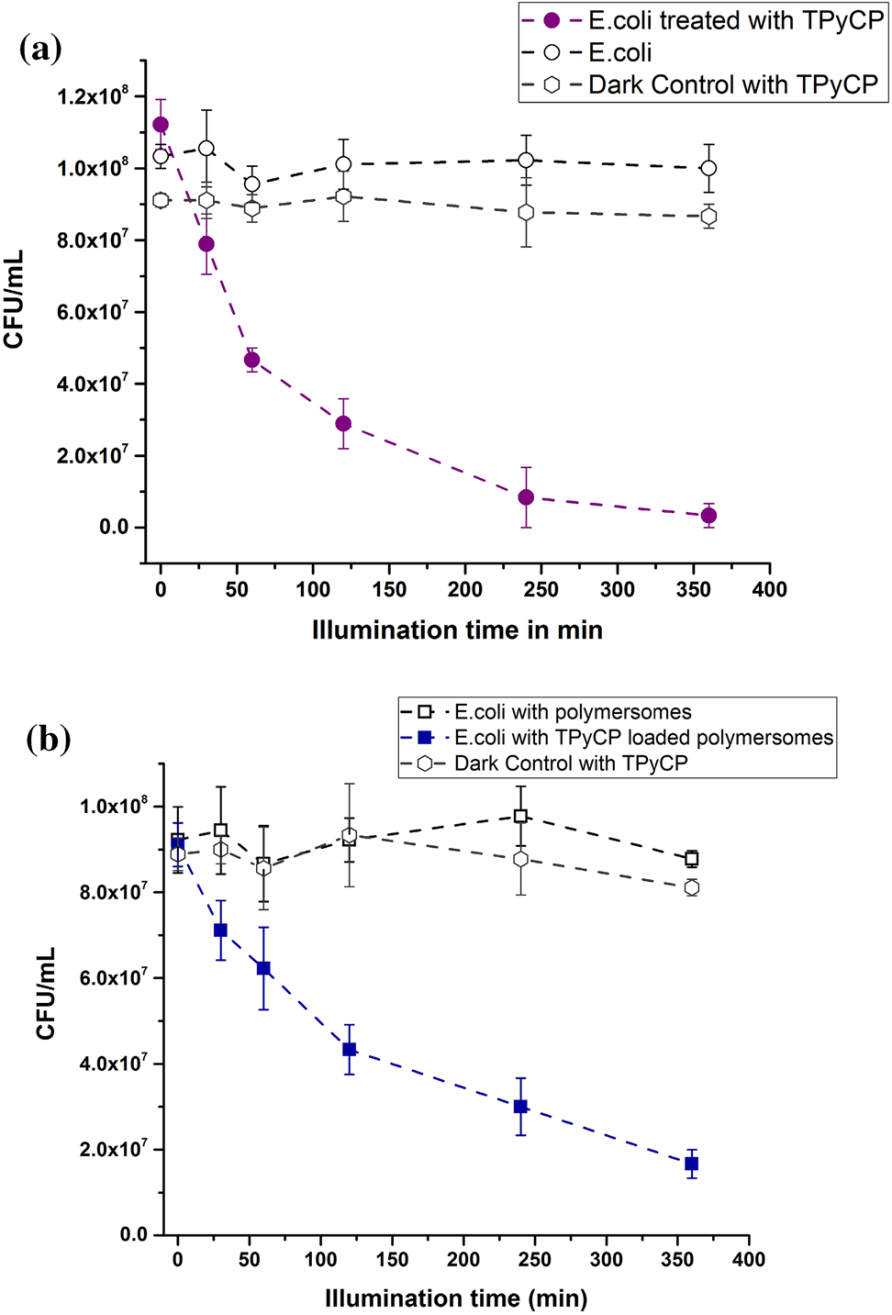
**a** CFU *E. coli* when the bacteria were irradiated with a LED red source (*λ*_max_ = 660 nm) and treated with 200 μΜ free TPyCP (purple circles), not treated with TPyCP (circle), and without irradiation in presence of TPyCP (hexagons). **b** CFU of *E. coli* treated with: TPyCP-loaded polymersomes irradiated with a LED red source (*λ*_max_ = 660 nm) (blue squares), TPyCP-loaded polymersomes without irradiation (hexagons) and empty polymersomes irradiated with a LED red source (*λ*_max_ = 660 nm) (squares)

In order to test the bio-functionality of the TPyCP-loaded polymersomes, we evaluated their ability to inhibit or prevent the growth of bacteria using a previously established protocol [48]. We determined the correlation between the colony-forming units (CFU) of *E. coli* and free TPyCP (200 μΜ in D_2_O) or TPyCP-loaded polymersomes (200 μM in D_2_O), respectively, upon irradiation and in dark conditions (Fig. 8). The oxidative stress due to irradiation of the free porphyrin caused a CFU reduction of 31% after 30 min of irradiation, and up to 94% after 360 min (Fig. 8a). Under the same red LED light irradiation, liquid cultures in the absence of porphyrin or porphyrin-loaded polymersomes were unharmed (Fig. 8a). The bacterial population remained stable for a period of at least 360 min at room temperature under red light irradiation. The viability of *E. coli* was not affected either by irradiation or by the substrates and oxidation products related to the experiments.

Similar behaviour was observed for bacteria cultures treated with porphyrin-loaded polymersomes: they were able to drastically reduce CFU values only upon irradiation (Fig. 8b). As expected, in the presence of empty polymersomes no influence on bacterial growth upon irradiation was detected. Furthermore, porphyrin-loaded polymersomes had no effect on the CFU in the dark, which supports an “on demand” functionality: only upon irradiation can the porphyrin-loaded polymersomes produce singlet oxygen inducing significant bacterial inhibition. The porphyrin-loaded polymersomes remain “silent” in the dark. Porphyrin-loaded polymersomes induced a significant decrease of the CFU in a rapid manner.

### Live/Dead^®^ staining

**Fig. 9:**
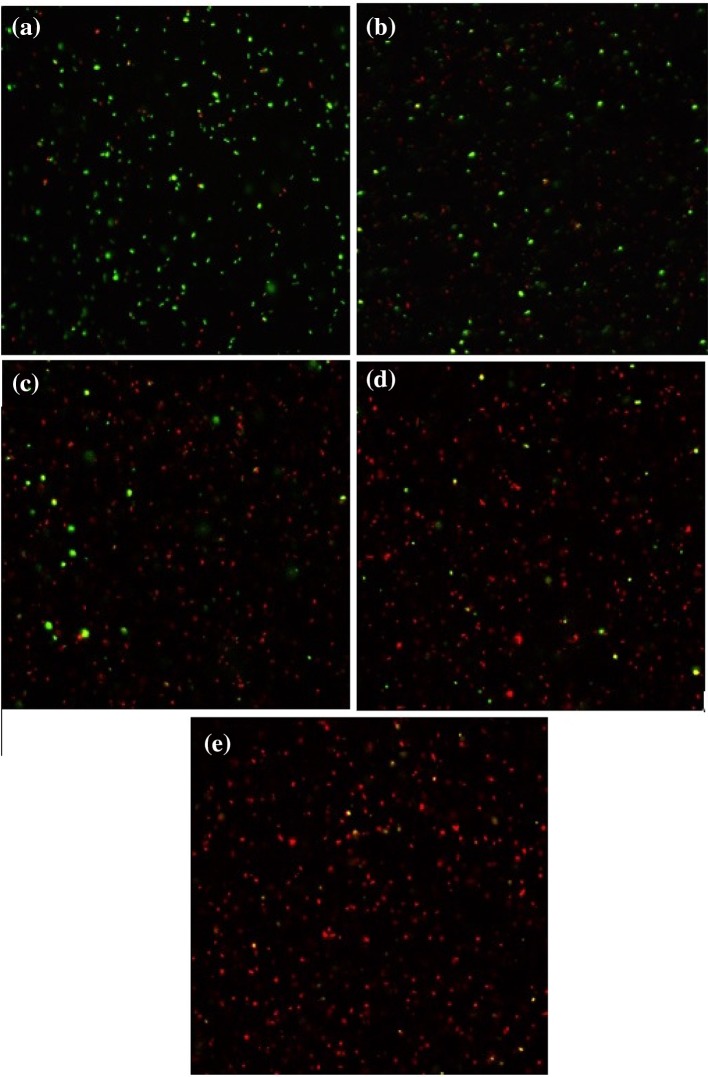
*E. coli* bacteria stained with SYTO 9 (considered alive, green) and propidium iodide (considered dead, red) incubated in presence of 200 μΜ free TPyCP after 0 min (**a**), 30 min (**b**), 120 min (**c**), 240 min (**d**) and 360 min (**e**) of illumination under red LED light (*λ*_max_ = 660 nm)

**Fig. 10:**
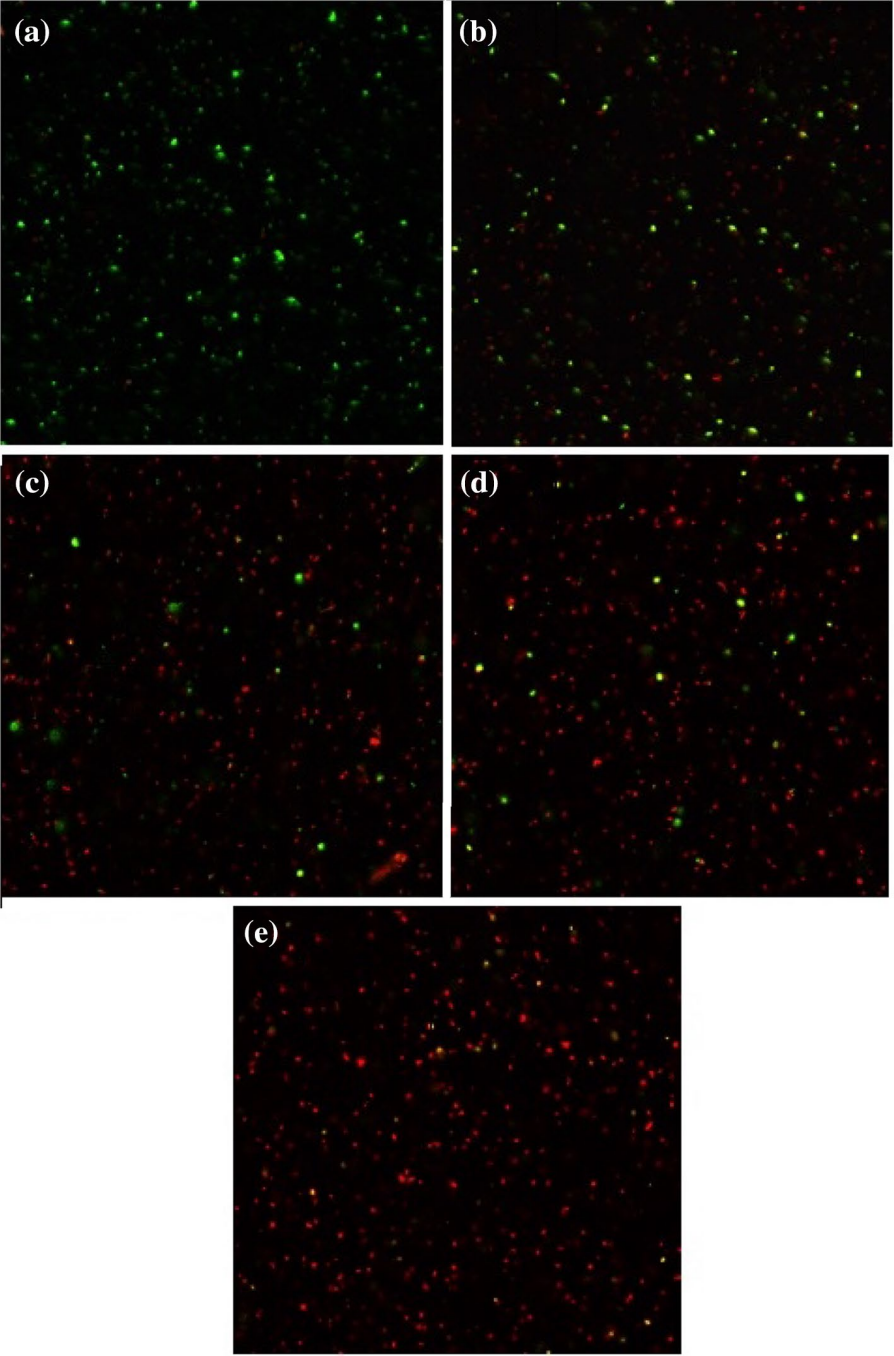
*E. coli* bacteria stained with SYTO 9 (considered alive, green) and propidium iodide (considered dead, red) incubated in presence of TPyCP-loaded polymersomes after 0 min (**a**) 30 min, (**b**) 120 min, (**c**) 240 min, (**d**) and 360 min (**e**) of illumination under red LED light (*λ*_max_ = 660 nm)

Another qualitative approach to study the bio-functionality of the free TPyCP and of the porphyrin-loaded polymersomes was to stain *E. coli* cultures and visualize them as described above in materials and methods section (Figs. 9, 10). This allows the qualification of the living-to-dead cell populations. The liquid cultures were exposed to the same oxidizing conditions, with the only difference being the staining step based on a 1:1 mixture of SYTO 9 green-fluorescent nucleic acid stain and red-fluorescent nucleic acid stain (propidium iodide). These stains differ both in their spectral characteristics and in their ability to penetrate healthy bacterial membranes. When used alone, SYTO 9 stain generally labels all bacteria in a population, i.e., those with intact membranes and those with damaged membranes. In contrast, propidium iodide penetrates only bacteria with damaged membranes, causing a reduction in SYTO 9 stain fluorescence when both dyes are present. Therefore, bacteria with intact cell membranes (considered alive) stain fluorescent green, whereas apoptotic bacteria stain fluorescent red. The *E. coli* culture treated with 200 μΜ TPyCP initially contained mainly alive bacteria (green). After 30 min of irradiation of the free porphyrin, the number of live bacteria had reduced, and after 240 min of irradiation (Fig. 9d) the difference is clearly visible, with most of the bacteria staining red. Singlet oxygen killed almost all of the *E. coli* population within 360 min of irradiation (Fig. 9e).

Of greater significance is what happens when photoactivation of TPyCP takes place within polymersomes (Fig. 10). Similar to the free porphyrin, at the beginning the bacteria are alive (green stain), while as the irradiation time increases the population shifts towards a red stain. After 360 min of irradiation (Fig. 10e), almost all bacteria were apoptotic (red). Once more, as indicated from the study of CFU, only the combination of irradiation and TPyCP is able to damage *E. coli.* When treated with TPyCP, but kept in dark, and as well as when irradiated, but in absence of TPyCP, the bacteria stay unharmed for 360 min (Figs. S4–S7).

## Conclusions

A tetraalkylpyridinium porphyrin TPyCP has been prepared, with the aim of exploiting its light absorption for photosensitized conversion of triplet to singlet oxygen. The presence of the set of four *Q* bands allows the compound to operate in the deep red, with an excitation wavelength as high as 660 nm. The compound is a photocatalyst for the oxidation of l-methionine and, more importantly, its activity is not diminished by encapsulation in polymersomes. Although the encapsulated TPyCP remains internalized in the polymersome, the small, long-lived and reactive singlet oxygen can diffuse through the membrane and react with external substrate. Encapsulation allows incubating the compound with a bacterial culture, without the drawback of the photosensitizer diffusing in the media. Live bacteria decrease significantly when the TPyCP-loaded polymersomes are irradiated with red light. These promising results prove the antimicrobial activity of TPyCP-polymersome system and make us consider expanding the biological evaluation towards in vitro studies on human cells.

## Electronic supplementary material

Below is the link to the electronic supplementary material.

Supplementary material 1 (PDF 2187 kb)

## Abbreviations

PDT: Photodynamic therapy
PS: Photosensitizer
LS: Light scattering
TEM: Transmission electron microscopy
LSM: Laser scanning microscopy
CFU: Colony-forming units
CLSM: Confocal laser scanning microscopy
LB: Lysogeny broth
PBS: Phosphate buffered saline
ROS: Reactive oxygen species
TSB: Tryptic soy broth
ESI: MS electrospray ionization mass spectrometry
LED: Light-emitting diode
TPP: 5,10,15,20-Tetraphenylporphyrin
TPyP: 5,10,15,20-Tetra(pyridin-4-yl)porphyrin
TPyCP: 5,10,15,20-Tetrakis(1-(6-ethoxy-6-oxohexyl)-4-pyridin-1-io)-21*H*,23*H*-porphyrin tetrabromide
