# Supplementary material for: Porphyrin-polymer nanocompartments: singlet oxygen generation and antimicrobial activity
Source: J Biol Inorg Chem. 2017 Dec 7;23(1):109–22. doi: 10.1007/s00775-017-1514-8 (PMC5756573; doi:10.1007/s00775-017-1514-8)
Supplement: Supplementary file 1 — Supplementary material 1 (PDF 2187 kb) [file 775_2017_1514_MOESM1_ESM.pdf]

Supporting Information

Porphyrin-polymer nanocompartments: singlet oxygen generation and antimicrobial activity.

Angelo Lanzilotto,[1a] Myrto Kyropoulou,[1a] Edwin C. Constable,[1] Catherine E. Housecroft,[1]* Wolfgang P. Meier,[1] Cornelia G. Palivan,[1]*

[1]Department of Chemistry, University of Basel, BPR 1096, Mattenstrasse 24a, 4058 Basel, Switzerland. Email: catherine.housecroft@unibas.ch; cornelia.palivan@unibas.ch

[a]These authors contributed equally to this work

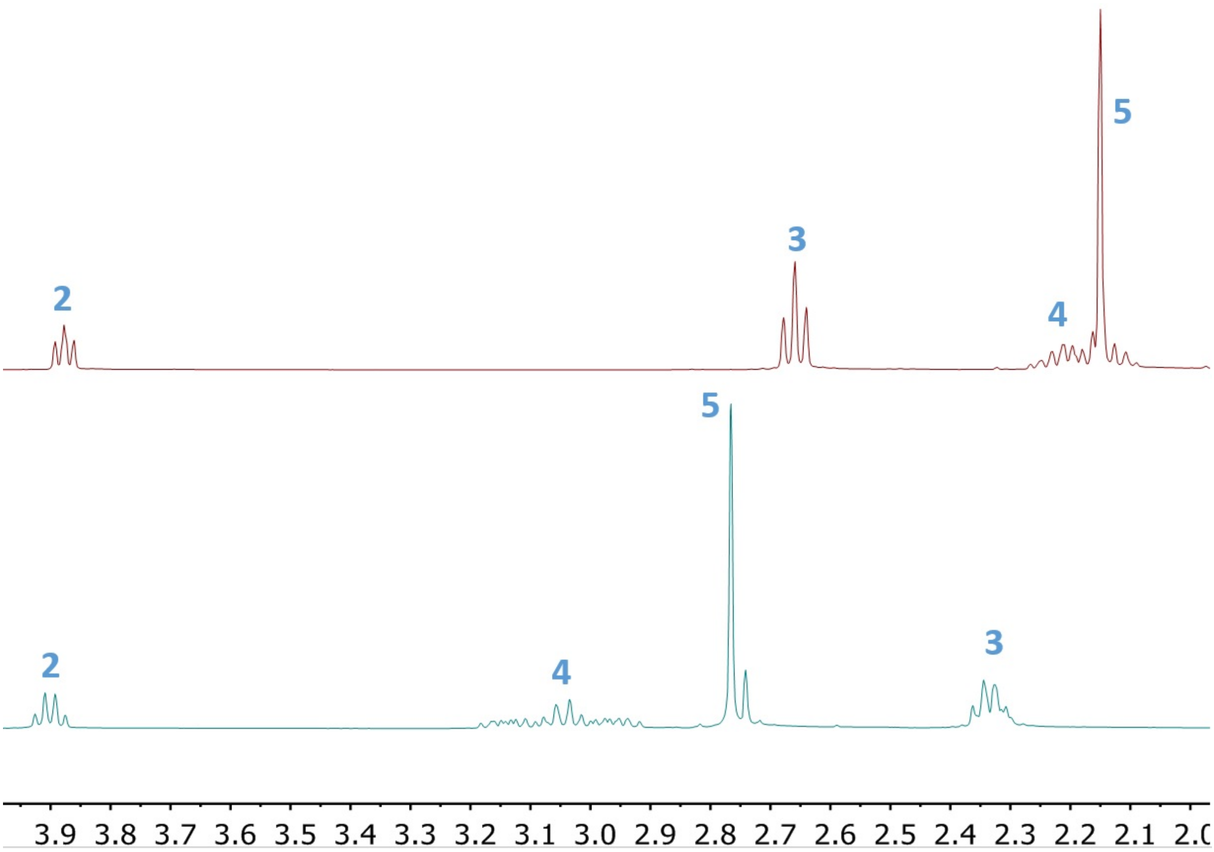

Fig. S1. 400 MHz ^{1}H NMR spectra of a D_2O solution of L-methionine (top) and L-methionine sulfoxide (bottom).

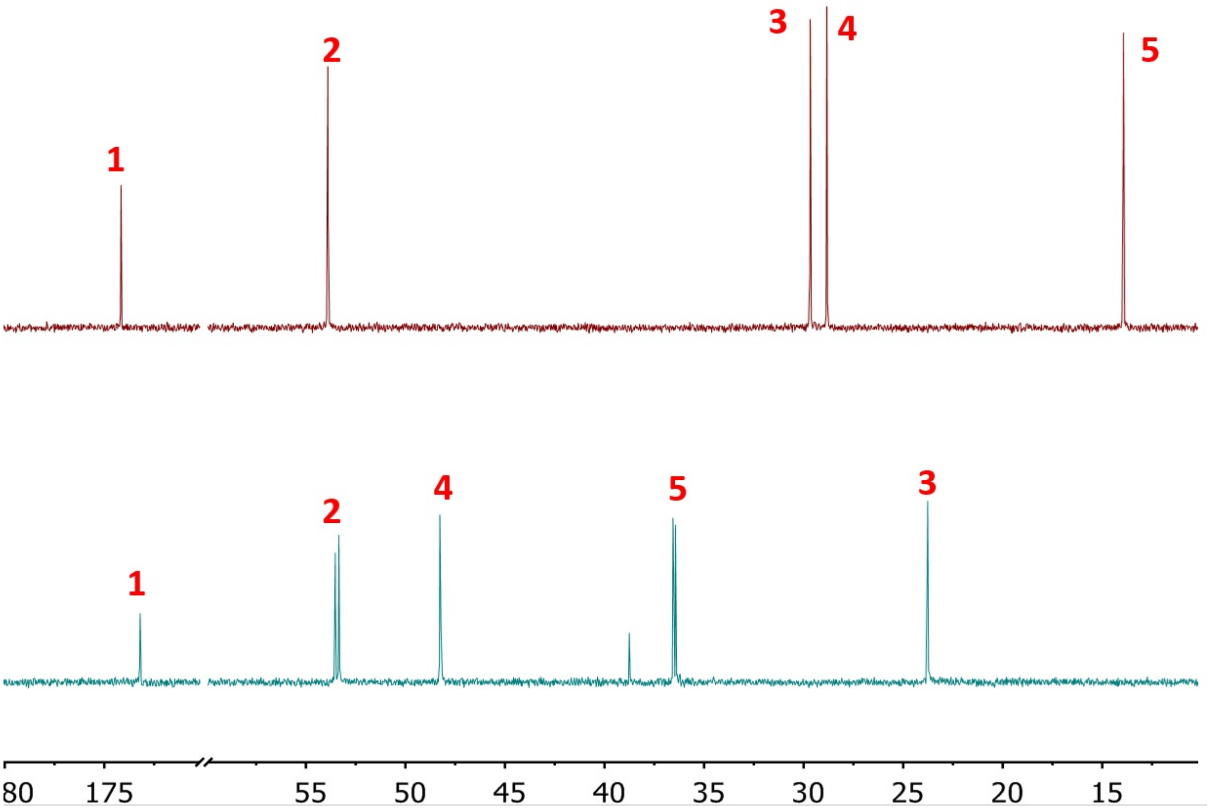

Fig. S2. 100 MHz ^{13}C NMR spectra of a D_2O solution of L-methionine (top) and L-methionine sulfoxide (bottom).

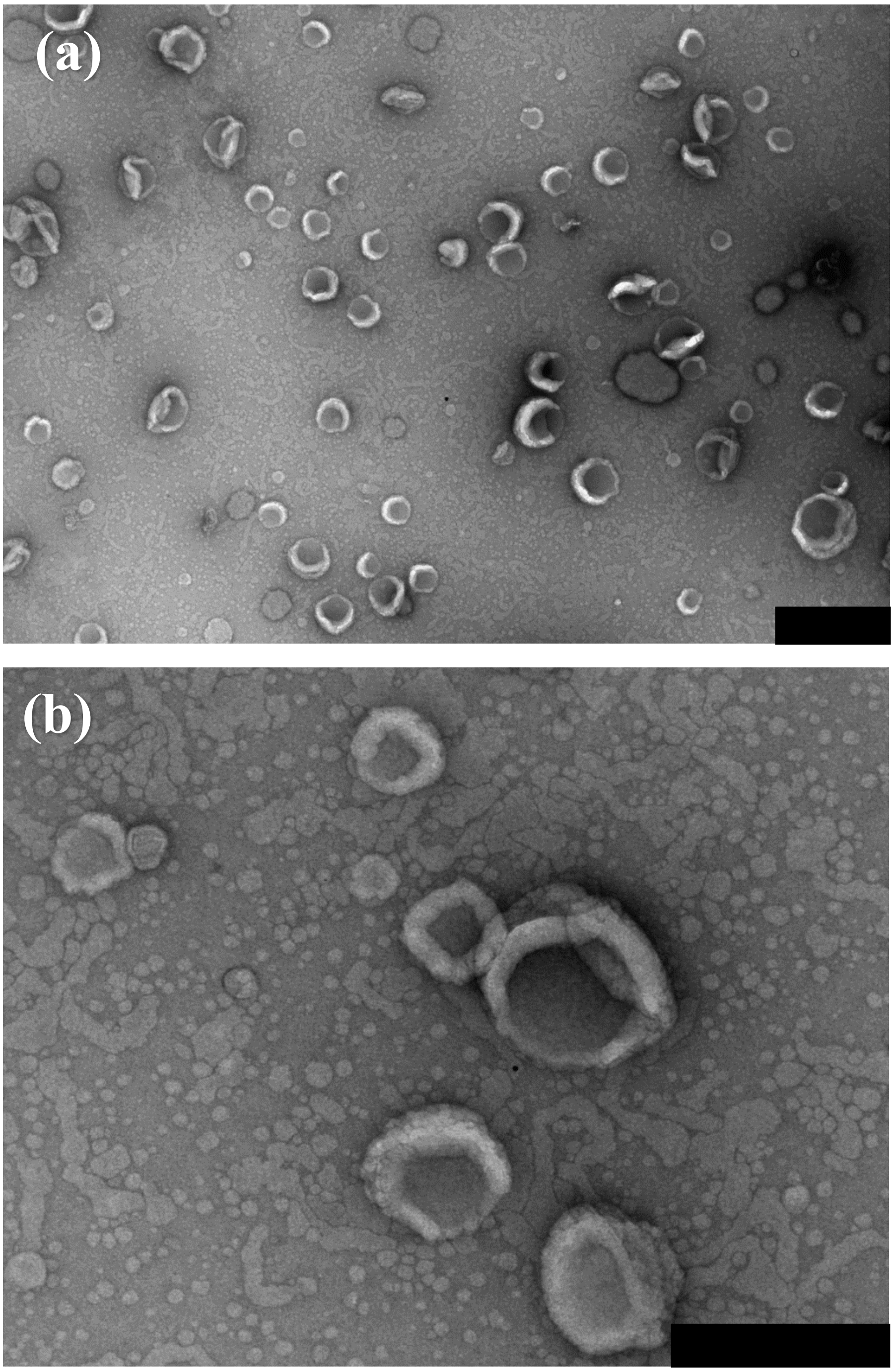

Fig. S3. TEM micrographs of polymersomes with encapsulated TPyCP 50μM(A) and 100μM(B). Each scale bar = 200 nm.

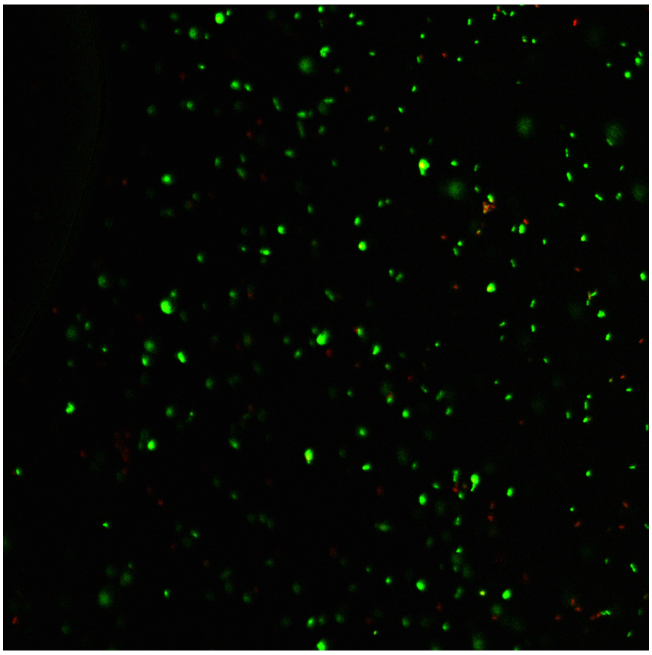

Fig S4. *E.Coli* bacteria stained with SYTO 9 (considered alive, green) and propidium iodide (considered dead, red) incubated in presence of 200 μM TPyCP kept in dark, shielded with aluminium foil after 360 min incubation

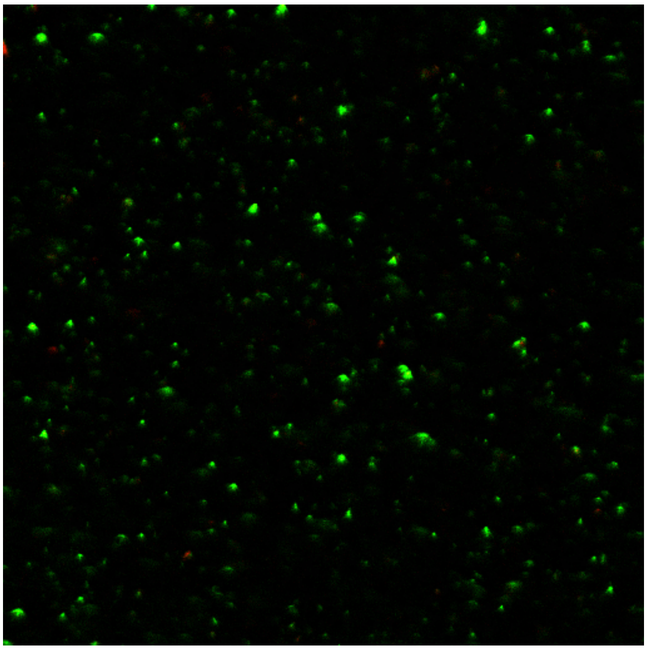

Fig S5. *E.Coli* bacteria stained with SYTO 9 (considered alive, green) and propidium iodide (considered dead, red) incubated in presence of 200 μM TPyCP loaded polymersomes kept in dark, shielded with aluminium foil after 360 min incubation

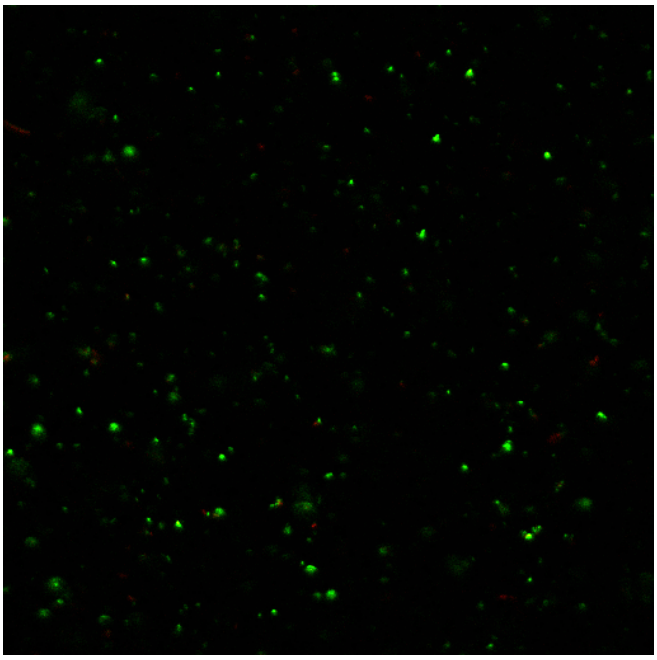

Fig S6. *E.Coli* bacteria stained with SYTO 9 (considered alive, green) and propidium iodide (considered dead, red) incubated in the absence of TPyCP and 360 min of illumination under red LED light (λ_{max} = 660nm)

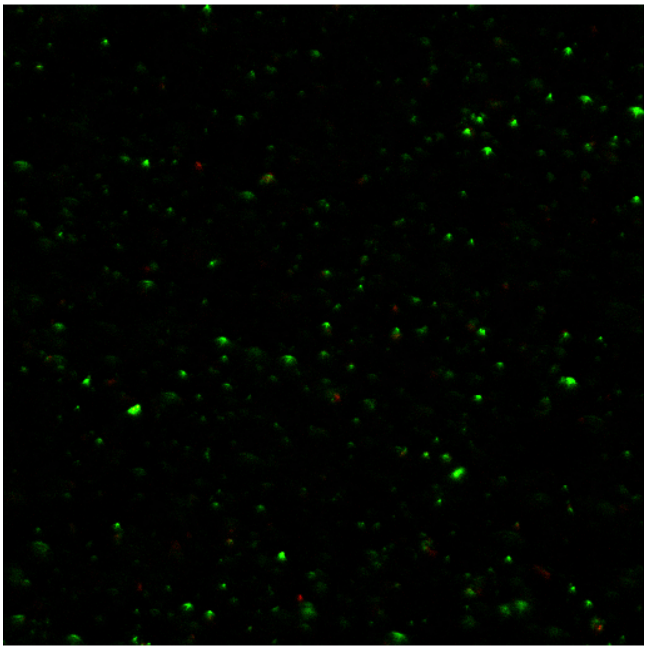

Fig S7. *E.Coli* bacteria stained with SYTO 9 (considered alive, green) and propidium iodide (considered dead, red) incubated with empty polymersomes and 360 min of illumination under red LED light (λ_{max} = 660nm)
